# A new role for Rrm3 in repair of replication-born DNA breakage by sister chromatid recombination

**DOI:** 10.1371/journal.pgen.1006781

**Published:** 2017-05-05

**Authors:** Sandra Muñoz-Galván, María García-Rubio, Pedro Ortega, Jose F. Ruiz, Sonia Jimeno, Benjamin Pardo, Belén Gómez-González, Andrés Aguilera

**Affiliations:** Centro Andaluz de Biología Molecular y Medicina Regenerativa-CABIMER, Universidad de Sevilla-CSIC-Universidad Pablo de Olavide, Seville, Spain; Duke University, UNITED STATES

## Abstract

Replication forks stall at different DNA obstacles such as those originated by transcription. Fork stalling can lead to DNA double-strand breaks (DSBs) that will be preferentially repaired by homologous recombination when the sister chromatid is available. The Rrm3 helicase is a replisome component that promotes replication upon fork stalling, accumulates at highly transcribed regions and prevents not only transcription-induced replication fork stalling but also transcription-associated hyper-recombination. This led us to explore the possible role of Rrm3 in the repair of DSBs when originating at the passage of the replication fork. Using a mini-*HO* system that induces mainly single-stranded DNA breaks, we show that *rrm3*Δ cells are defective in DSB repair. The defect is clearly seen in sister chromatid recombination, the major repair pathway of replication-born DSBs. Our results indicate that Rrm3 recruitment to replication-born DSBs is crucial for viability, uncovering a new role for Rrm3 in the repair of broken replication forks.

## Author summary

DNA replication needs to be precise to ensure cell survival and to avoid genetic instability. Different DNA obstacles, such as those originated by transcription, frequently hamper replication fork progression leading to fork stalling or even fork breakage. This requires the homologous recombination machinery to repair the damage. Here, we uncovered a role for yeast Rrm3, a replisome component known to promote replication upon fork stalling, in the repair of replication-born double strand breaks. In particular, *rrm3*Δ cells show a defect in the recombination with the sister chromatid, the preferred template for the maintenance of genome integrity. Our results support the possibility that the known accumulation of Rrm3 at sites of active transcription reflects an active role of Rrm3 in the repair of broken forks.

## Introduction

Genetic instability is a hallmark of cancer cells [1]. In the last few decades, evidence has shown that replication is one main source of genetic instability. Replication fork (RF) progression is hindered by the encounter with DNA obstacles such as protein-DNA complexes, damaged DNA or DNA breaks. In particular, the occurrence of transcription creates an important source of RF stalling [2]. If a stalled RF is processed by endonuclease cleavage or if the replisome encounters a nick or single stranded DNA gap, it can give rise to DNA double-strand breaks (DSBs), one of the most cytotoxic DNA lesions. In eukaryotic cells, DSBs can be repaired either by non-homologous end joining (NHEJ) or by homologous recombination (HR), which is used preferentially in the S/G2 phases of the cell cycle when the sister chromatid is available. Sister chromatid recombination (SCR) is, thus, the preferred mechanism to ensure the maintenance of genome integrity [3–5]. Specific factors have been involved in the choice of the sister chromatid as the template for the repair of replication-born DSBs, such as the Smc5-6 complex [5, 6] or the acetylation state of Histone H3 lysine 56 residue [7].

Rrm3 was discovered as an inhibitor of HR between the ribosomal DNA and *CUP1* tandem direct repeats [8]. All sites affected by the absence of Rrm3 are assembled into non-nucleosomal protein-DNA complexes implying that Rrm3 acts directly or indirectly to facilitate replication through protein-DNA complexes [9–12]. The accumulation of Rrm3 at highly transcribed regions as a consequence of RF stalling [13] suggests that Rrm3 might have a role in the progression of stalled RFs but no evidence has been reported on whether Rrm3 is required for repair of transcription-associated damage. Indeed, Rrm3 has been shown to prevent not only transcription-induced RF stalling but also transcription-associated hyper-recombination [14]. *RRM3* has a reported negative genetic interaction with many genes involved in HR [15] as well as with the *rem* specific type of *rad3/XPD* Nucleotide Excision Repair (NER) mutation of TFIIH, *rad3-102* [16], which blocks NER at a post-incision intermediate and causes an extended retention of TFIIH at the damaged DNA, channelling bulky adducts to DSBs when reached by the RF [17]. The increased levels of HR in the absence of Rrm3 in certain DNA regions such as the rDNA [8] advocated Rrm3 as an anti-recombinase at stalled RFs similar to Srs2 [18, 19]. Indeed, Rrm3 is required for the normal growth of cells that have a functional HR pathway when either Sgs1 or Srs2 are absent [15, 19]. The weak DNA damage sensitivity of *rrm3*Δ cells, however, has suggested that Rrm3 does not play a critical role in DNA damage repair [15]. Here we have analysed the role of Rrm3 in different genetic and molecular HR systems after the induction of RF breakage. Notably, we uncover an unexpected role for Rrm3 in the recombinational repair of replication-born DSBs, in particular that occurring with the sister chromatid, the primary event required after RF breakage.

## Results

### Rrm3 is required for the recombinational repair of replication-born DSBs

**Fig 1:**
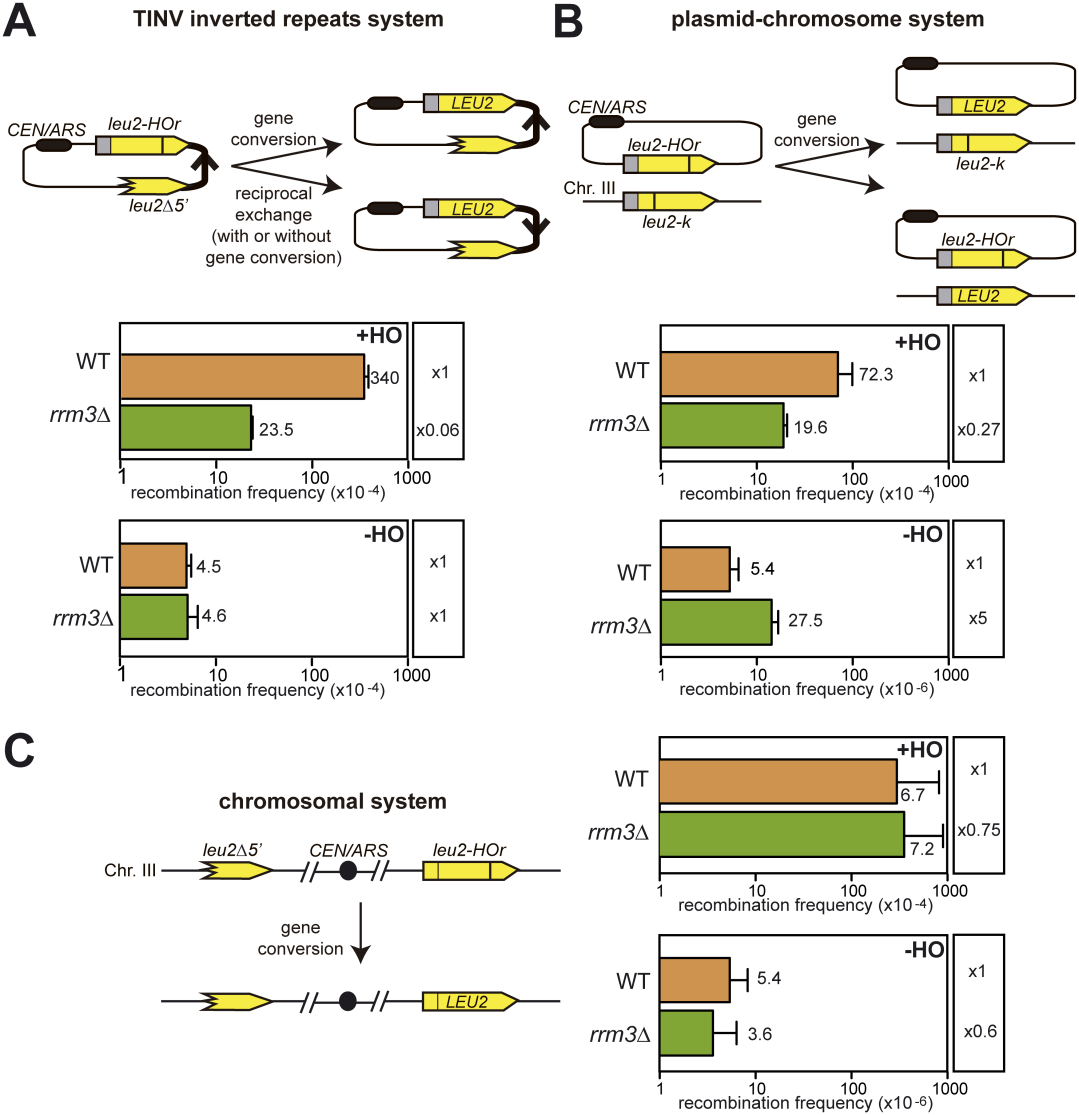
Genetic analyses of spontaneous and HO-induced recombination. (A) Analysis of Leu+ recombination events with the TINV plasmid recombination system, which measures recombination between the two inverted *leu2*Δ*5’ and leu2-HOr* repeats of the pTINV plasmid (B) Analysis of Leu+ recombination events with the plasmid-chromosome recombination system that measures recombination between the *pCM189-L2HOr* plasmid and the chromosomal *leu2-k* allele (C) Analysis of Leu+ recombination events with the chromosomal system that measures recombination between the *leu2*Δ*5’ and leu2-HOr* repeats located on either arm of chromosome III. All experiments were performed in wild-type and *rrm3*Δ cells either after HO activation in 2% galactose (+HO) or without HO activation to measure spontaneous events (-HO). Diagrams of systems are shown above each graph. Values plotted for each genotype are the average and SD of the median values of three fluctuation tests (each based on 6 samples) performed with three independent transformants in the case of the TINV and plasmid-chromosome recombination systems and the average and SD of 6 independent colonies in the chromosomal system.

The use of a minimal 24-bp endonuclease *HO* site (mini *HO*r) [20] provides a unique tool to mimic a natural situation in which DSBs appear as a consequence of replication failures [6, 21]. At this site, the HO endonuclease produces mainly single-stranded DNA nicks on any of the strands that are converted into DSBs when they are encountered by the RFs [6]. We performed genetic analysis to compare the ability to repair these HO-induced replication-born DSBs versus spontaneous DSBs (Fig 1). We performed these analyses with different systems in which the two homologous sequences were located in the same molecule, either in the same plasmid (TINV system, Fig 1A) or in the same chromosome (Fig 1C); or on different molecules, one in a plasmid the other in a chromosome (plasmid-chromosome recombination, Fig 1B). The TINV system (Fig 1A) allows genetic detection of HO-induced replication-born DSB repair occurring by several mechanisms: equal and unequal mainly SCR, plus a small contribution of intrachromatid recombination [5, 6]. The chromosomal system, instead, detects only intermolecular recombination (Fig 1C). In addition, Leu+ recombinants can arise by either gene conversion or reciprocal exchange in the TINV system [21], and as a result of gene conversion in the chromosomal system (Fig 1C), since reciprocal exchange would lead to an unviable dicentric chromosome. Note that the *leu2-k* and the *leu2*Δ*5’* alleles used as donors of repair of the HO break are genetically equivalent for our purpose, since *leu2*Δ*5’* is truncated at the *Cla*I site, close by the *Kpn*I site mutated in *leu2-k* [5, 22]. Thus, the recombination events that can be genetically scored in the two systems cannot go beyond the *Kpn*I site to give a productive Leu+ recombinant.

After 5 hours of HO induction, we observed that *rrm3*Δ led to a 15-fold reduction in the TINV system, but only a 4-fold reduction in the plasmid-chromosome system and no significant reduction in the chromosomal system (Fig 1). Although Rrm3 must be involved in other repair processes, it seems clear that recombination with the sister chromatid is affected the most in the absence of Rrm3 given that the TINV assay is the only one that accounts for SCR.

By contrast, the absence of Rrm3 did not affect spontaneous recombination measured with the TINV plasmid construct or with the chromosomal systems, whereas it led to a 5-fold increase in plasmid-chromosome recombination (Fig 1). Such increase is in agreement with the hyper-recombination phenotype of *rrm3*Δ previously reported in some assays such as recombination between homologous chromosomes [23].

In our constructs, the low efficiency of this *HOr* site allows the cleavage of only one of the sister chromatids, the other one remaining intact in most cases and competent to be used as a template [5]. The fact that we only observe a decreased repair frequency in *rrm3*Δ cells after HO-induced cleavage and not in spontaneous conditions suggests that Rrm3 is involved in the repair of replication-born DSBs.

### Rrm3 is not required for the repair of replication-independent DSBs or for break-induced replication

**Fig 2:**
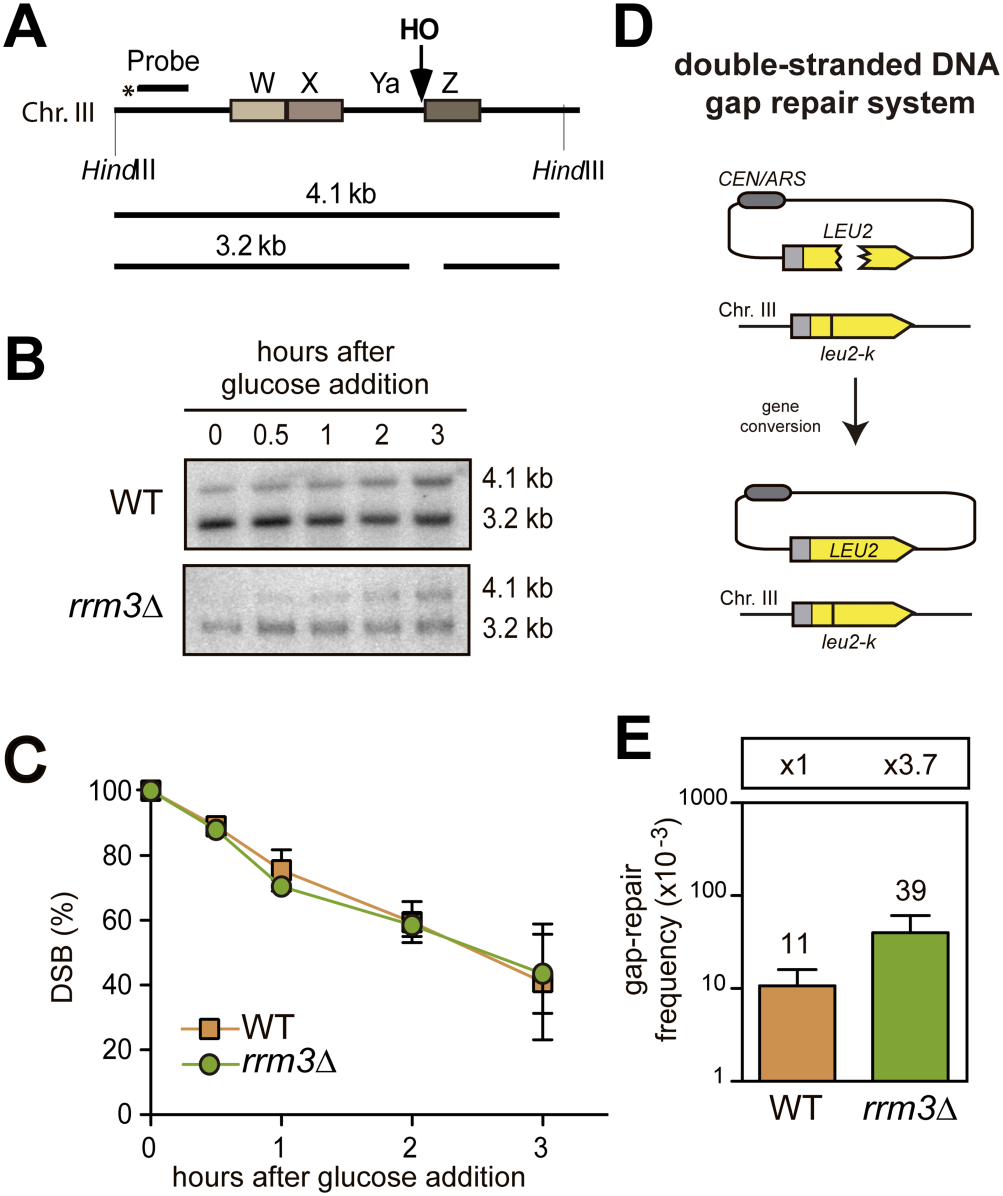
Analysis of the repair of replication-independent DSBs. (A) Scheme of the *MAT***a** region on chromosome III and the fragments obtained after digestion with *Hind*III. (B) DSB repair in isogenic wild-type and *rrm3*Δ strains. Strains were incubated in 2% galactose media to induce HO and the analysis of repair was performed at the indicated time points after glucose addition. *Hind*III-digested genomic DNA was analysed by Southern-blot with the *MAT* specific probe depicted in A. A representative blot is shown. (C) Quantification of the percentage of DSBs at different times after glucose addition. The average and SD of three independent experiments is shown. (D) Double-stranded DNA gap repair system. The centromeric ARS-containing *pCM189-L2HOr* plasmid with a double-strand gap in *LEU2* can be repaired from the homologous chromosomal *leu2-k* sequence. (E) Average recombination frequency and SD of three independent experiments in the indicated strains.

To determine if the absence of Rrm3 leads to defective repair of a replication-independent DSB, the repair of DSBs arising from the cleavage of the endogenous full *HO* site present in the wild-type *MAT* locus on chromosome III was analysed by Southern-blot hybridization with a *MAT* specific probe (Fig 2A). The *HO* cleavage obtained after 2 hours of growth in a galactose of cells that had been transformed with a plasmid containing the HO endonuclease gene under the *GAL1* promoter reached up to 95% in both wild-type and *rrm3*Δ cells. After 3 hours in galactose, when cells had not divided yet (S1A Fig), this initial DSB signal was reduced to 40% in either wild-type or *rrm3*Δ cells (Fig 2B and 2C). This result indicates that HO-induced DSBs can be efficiently repaired even in the absence of Rrm3. In this assay, homology-mediated repair would occur by gene conversion with *HML* or *HMR* as a donor. Since our measurements were taken in asynchronous cultures, some repair events could also be due to NHEJ.

To further confirm the specificity of Rrm3 for replication-dependent DSBs, we assayed the repair of a double-stranded DNA gapped plasmid. For this purpose, the *pCM189*-*L2HOr* plasmid was digested with *Mfe*I. Its introduction into host cells carrying the *leu2-k* mutation allows homology-dependent repair, which can be quantified by counting colony-forming units in SC -Leu -Ura (Fig 2D, see Materials and methods). In this media, NHEJ and reciprocal exchange events cannot be detected, because either they do not lead to Leu^+^ Ura^+^ colonies or result into unstable dicentric chromosomes, respectively. Therefore, only Leu^+^ Ura^+^ gene conversion events can be detected. The *rrm3*Δ mutation did not cause any decrease in the frequency of gap repair, confirming that Rrm3 is specifically involved in the recombinational repair of replication-born DSBs. Actually, the gap-repair frequency in *rrm3*Δ was of 3.9 x 10^−2^, 3.7-fold higher than wild-type levels (Fig 2E), implying a potential additional impact of Rrm3 inactivation in the origin of recombination events.

**Fig 3:**
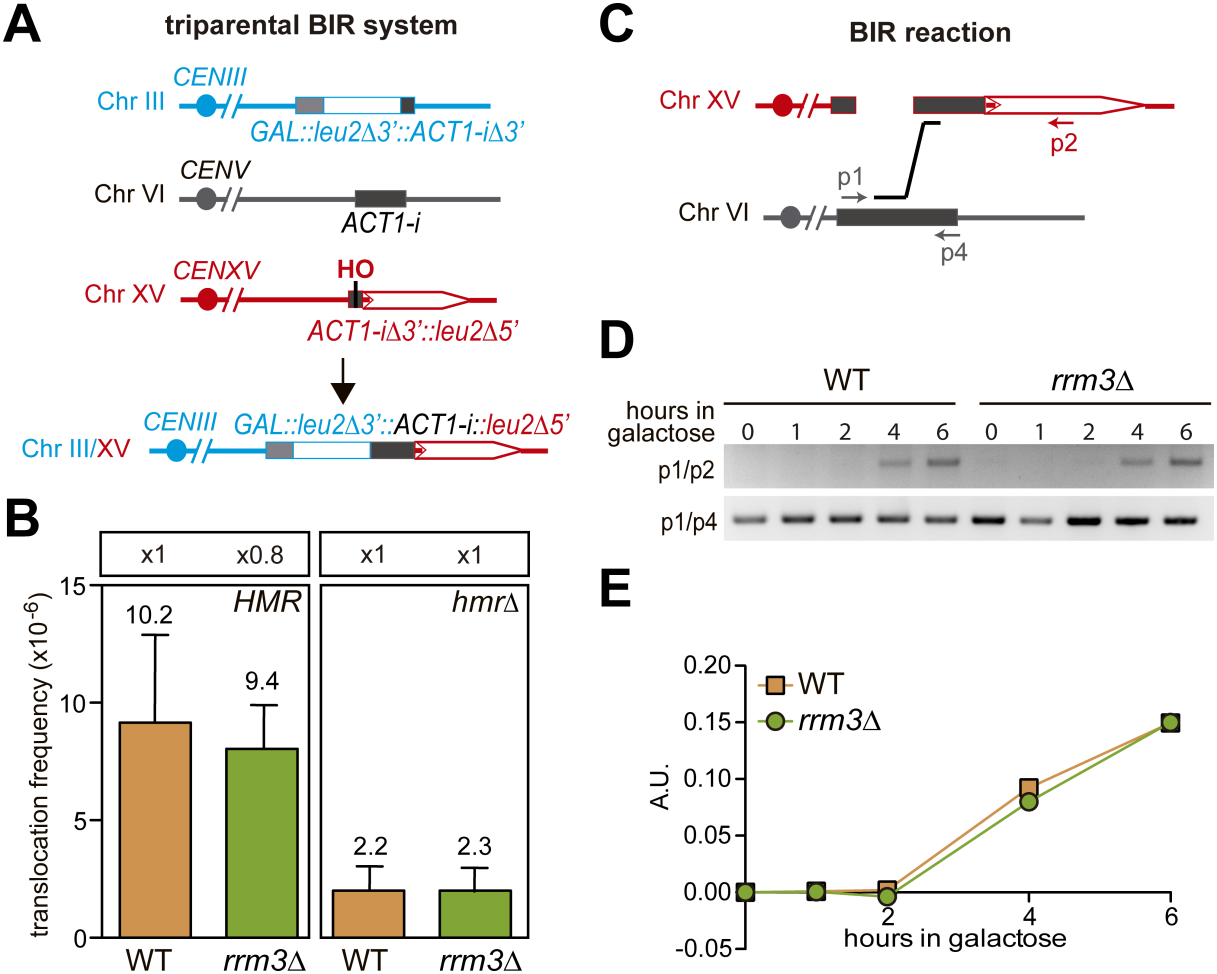
Analysis of BIR of replication-independent DSBs. (A) Triparental BIR system. In this system, the centromere-distal DSB end at chromosome XV is repaired by a triparental BIR reaction that uses the homology with the endogenous ACT1 intron at chromosome VI as a bridge template. The centromere-proximal DSB end on chromosome XV has homology with both *HMR* and *MAT***a** sequences where it can initiate a second BIR reaction required for the complete repair of the DSB. (B) Frequency of triparental BIR after HO-induced DSBs in the indicated strains. Values plotted for each genotype are the average and SD of three independent experiments. (C) Schematic representation of the BIR reaction analysed and the primers used. (D) PCR detection of the BIR intermediate when the HO-cut chromosome III invades the homologous *ACT1* intron sequence on chromosome VI and control PCR product at the *ACT1* intron sequence. (E) PCR products were quantified, and values were normalized to those of the control.

Since broken RFs can also be repaired via break induced replication (BIR) [24] and the other yeast member of the Pif1 family of helicases, Pif1, but not Rrm3 is involved in BIR [25], we used a previously reported intron-based chromosomal translocation assay to study BIR. In this system, a DSB is generated by full *HO* cleavage in a single chromosome, XV, and can be repaired by a BIR-mediated triparental event (Fig 3A) [26]. In this event, the centromere-distal DSB end generated at chromosome XV uses the homology with the endogenous *ACT1* intron located at chromosome VI to initiate a first BIR event that serves as a bridge template to initiate a second BIR event with chromosome III giving rise to the Leu+ translocants measured (Fig 3A). The centromere-proximal DSB end on chromosome XV has homology with both HMR and *MAT***a** sequences with which it can initiate a second BIR reaction required for the complete repair of the DSB [26]. In the absence of the *HMR* locus, the translocation events are limited to those occurring with the *MAT***a** sequence. As shown in Fig 3B, the frequency of translocations was not significantly affected in *rrm3*Δ or *rrm3*Δ *hmr*Δ cells. The intermediates of the first single BIR reaction between chromosomes XV and VI were also detected at the molecular level by PCR after 4 hours of HO-induction both in wild-type and *rrm3*Δ cells (Fig 3C–3E). This is in agreement with Rrm3 not being involved in BIR [25].

Altogether, these results support the idea that Rrm3 is specifically involved in the recombinational repair of replication-born DSBs by a mechanism that does not involve BIR.

### Rrm3 is required for efficient sister chromatid exchange

**Fig 4:**
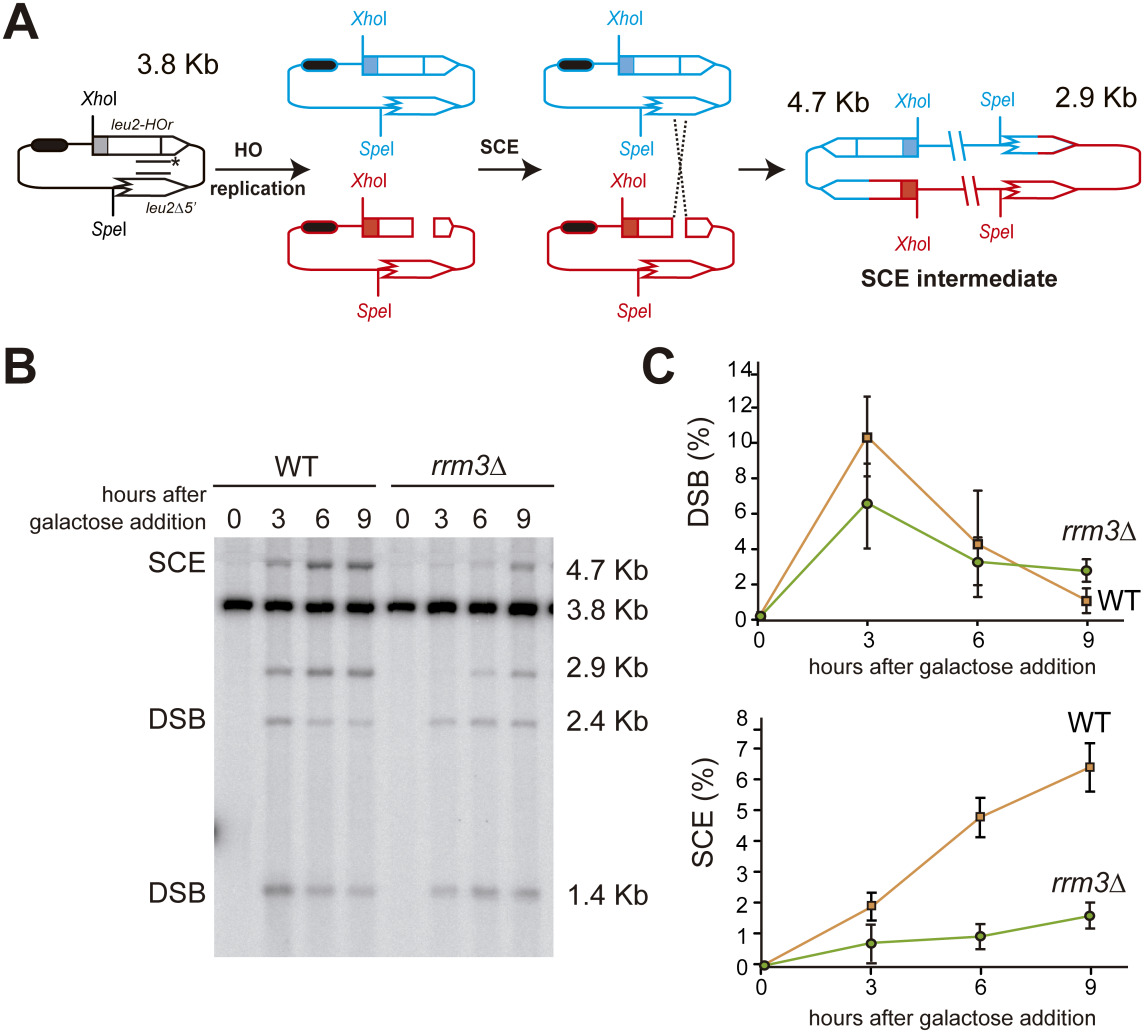
Physical analysis of SCE. (A) Schemes of the plasmid *pTINV* and the intermediates produced by SCE after HO-induced replication-born DSBs. The bands detected after *Xho*I-*Spe*I cleavage, using the *LEU2* probe (line with asterisks) are indicated with their corresponding sizes. (B) HO-induced formation of DSB and SCE intermediates in isogenic wild-type and *rrm3*Δ cells incubated in galactose for the indicated time points. *Xho*I-*Spe*I digested genomic DNA was analysed by Southern-blot with the *LEU2* probe depicted in A. A representative Southern blot is shown. The 3.8 Kb band corresponds to the intact plasmid and equal SCR events, the 1.4 Kb and 2.4 Kb fragments to the DSBs, the 2.9 Kb band can result from SCE and other processes such as break-induced replication and the 4.7 Kb band is specific for SCE. (C) Quantification of DSBs (1.4 Kb plus 2.4 Kb bands) and SCE (4.7 Kb band) relative to the total DNA. The average and SEM of three independent experiments is shown.

Given that the sister chromatid is the preferred template to repair broken replication forks [3–5], we decided to directly study the involvement of Rrm3 in SCR at the molecular level with the TINV inverted-repeat system (Fig 4A). When cells containing the HO endonuclease gene under the *GAL1* promoter grow in galactose media, replication-born DSBs can be observed by Southern-blot as 2.4 and 1.4 Kb bands [5, 21]. At the same time, DSB repair leads to the formation of new 4.7- and 2.9-Kb bands, the first of which is exclusively a consequence of unequal Sister Chromatid Exchange (SCE) events [5, 21]. It has been shown that this is an accurate indicator of the proficiency in total SCR [5, 6, 27]. Fig 4B shows the repair after 3, 6 or 9 hours of HO-induction in wild-type and *rrm3*Δ cells, when the culture had not completely duplicated once yet (S1B Fig). Whereas the 4.7-Kb band represented a 6% of the total DNA 9 hours after HO induction in wild-type cells, it barely reached 2% in *rrm3*Δ cells (Fig 4C) indicating a failure to repair with the sister chromatid in the absence of Rrm3.

### Rrm3 is required for survival after RF breakage

When we performed the recombination tests, we noticed that *rrm3*Δ did not affect the survival of replication-born DBSs at the mini-*HOr* site suggesting that the defect in SCR does not affect cell viability. However, this might be due to the low efficiency of mini-*HOr* cleavage, which is less than 10% with respect to the full 117-bp *HO* cleavage site [5]. Indeed, *rrm3*Δ cells are hypersensitive to camptothecin [28], which causes TopoI-linked single-stranded breaks. Paradoxically, *rrm3*Δ by itself is not sensitive to hydorxyurea (HU)-induced replication stress [15](S2 Fig), suggesting that RF breakage is not a frequent event in wild-type conditions even in the presence of such replicative stress.

**Fig 5:**
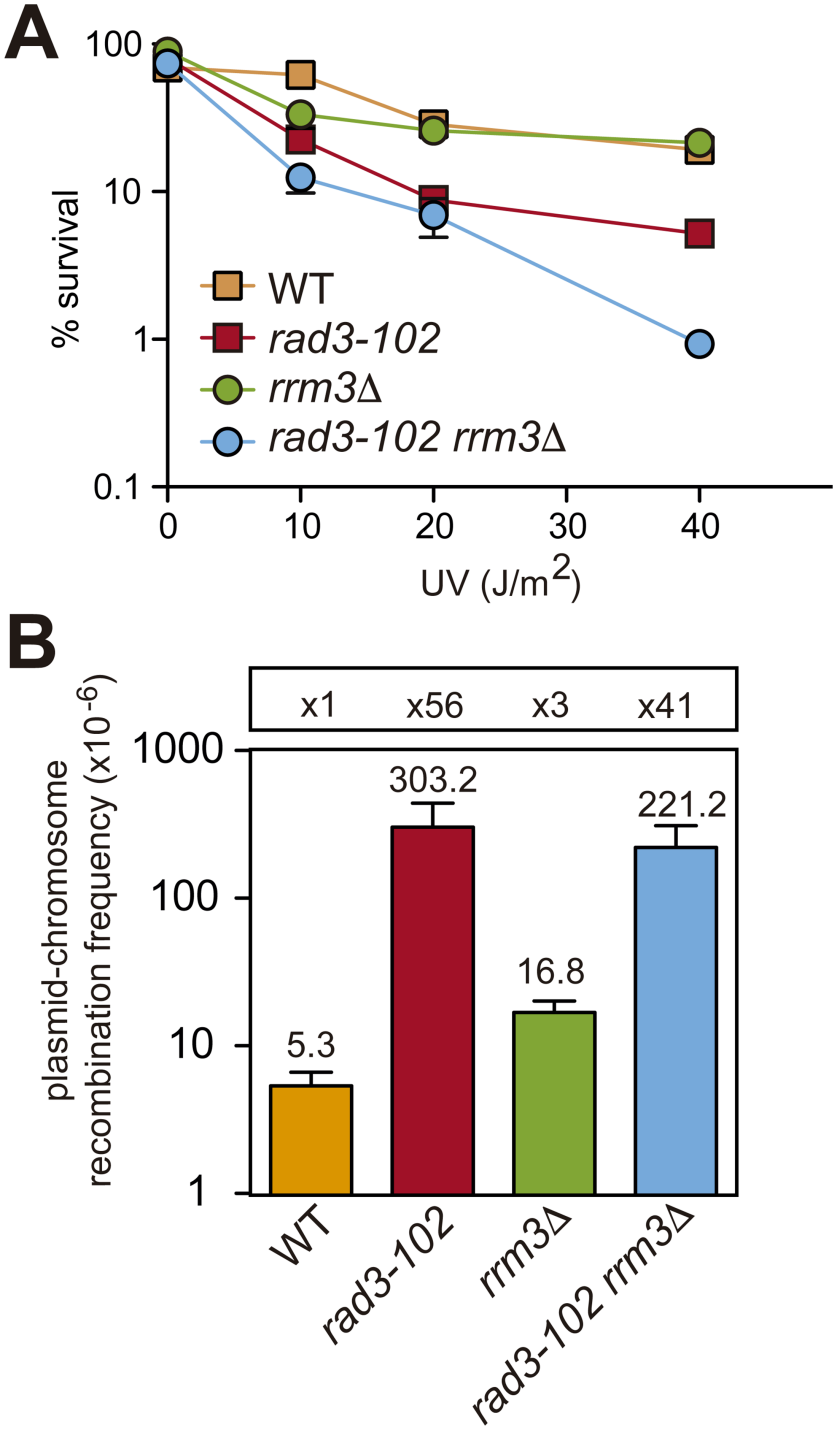
Genetic interaction of *rrm3*Δ with *rad3-102*. (A) Survival curves after exposure to UV-C of wild-type, *rrm3*Δ, *rad3-102* and *rad3-102 rrm3*Δ cells. Values plotted for each genotype are the average and SD of three independent experiments. (B) Analysis of spontaneous Leu+ plasmid-chromosome recombination events in the indicated strains. Values plotted for each genotype are the average and SD of the median values of three fluctuation tests (each based on 6 samples) performed with three independent transformants.

In order to determine the consequences of defective SCR on the viability of *rrm3*Δ, we decided to use a different genetic tool for the generation of replication-dependent DSBs, the *rad3-102* mutation, which impairs NER after the endonuclease cleavage step leading to a blocked TFIIH that can induce RF breakage [17]. To enhance the occurrence of such replication-induced breaks, we used increasing doses of UV radiation. As shown in Fig 5A and S2 Fig, *rrm3*Δ *rad3-102* is alive but showed enhanced sensitivity to UV suggesting that the high incidence of RF breakage after encountering a stacked TFIIH or abortive NER reaction at UV damaged sites is toxic in the absence of Rrm3 [17]. Consistently, the combination of *rrm3*Δ with *rad3-102* has also been reported to show enhanced sensitivity to the UV mimetic 4-NQO as well as to HU [17]. We confirmed these results and further observed an enhanced sensitivity of *rrm3*Δ *rad3-102* to methyl methanesulfonate (MMS) (S2 Fig).

Other mutants such as *pif1*Δ or *tof1*Δ have been reported to have similar genetic interactions with *rad3-102* [17]. We therefore analysed SCE in *pif1*Δ, *tof1*Δ and *rrm3*Δ transformed with a plasmid containing the HO endonuclease gene under the GAL1 promoter. Neither *pif1*Δ nor *tof1*Δ cells showed any significant defect in SCE as compared with *rrm3*Δ cells in the same background (S3 Fig). Therefore, such genetic interactions are probably explained by either the Pif1 role in BIR or the role of Tof1 in fork stabilization, as previously suggested [17].

In order to see if non-sister templates can compensate for the defective SCE, we also studied recombination levels with the plasmid-chromosome system (depicted in Fig 1B). As shown in Fig 5B, spontaneous recombination was not enhanced in *rad3-102 rrm3*Δ with respect to *rad3-102* cells. Together with lower survival, this suggests that non-sister templates cannot always substitute the sister chromatid to safely promote recombinational repair.

Altogether, our results support the conclusion that Rrm3 is specifically required for survival after RF breakage.

### Rrm3 enrichment at HO-induced DSBs depends on replication

**Fig 6:**
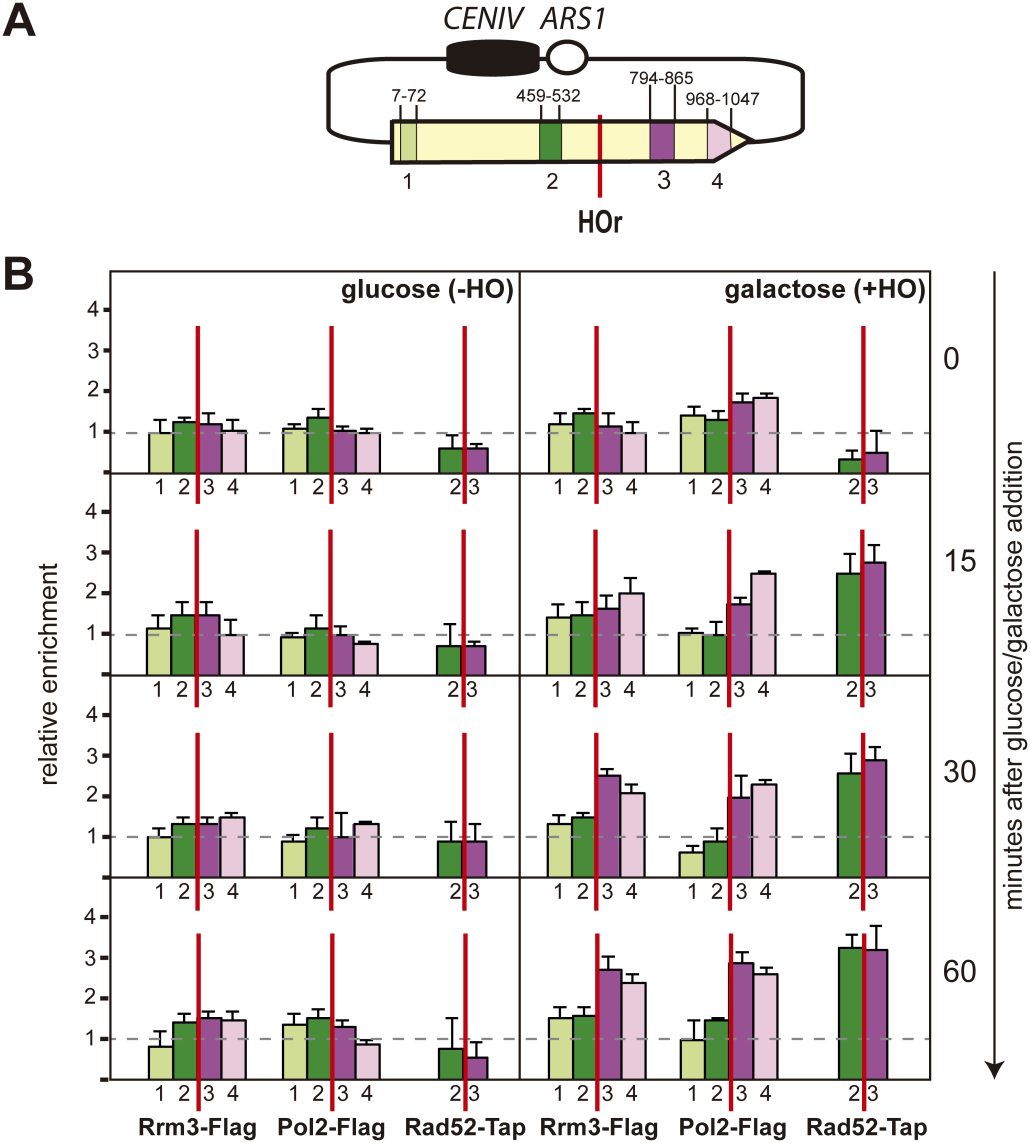
Analysis of the recruitment of Rrm3, Pol2 and Rad52 proteins to a replication-induced DSB. (A) A scheme of the *pCM189-L2HOr* analysed plasmid and the amplified PCR fragments, with the nucleotide positions from the *leu2-HOr* gene (B) ChIP analysis of Rrm3-FLAG, Pol2-TAP and Rad52-FLAG at the *leu2-HOr* allele. Samples were collected at different time points after glucose (-HO) or galactose (+HO) addition. The median and SD of three independent experiments is shown.

To assay the presence of Rrm3 on the DNA after RF breakage, we constructed Rad52-Flag, Pol2-Tap and Rrm3-Flag tagged yeast strains and used them to determine the presence of all proteins at DNA by chromatin immunoprecipitation (ChIP). This study was done at the *pCM189-L2HOr* plasmid before and after the induction of the HO endonuclease (Fig 6). Despite the distance, from the mini-*HOr* cleavage site to the *ARS* in this plasmid, being similar on both sides of the mini-HO cleavage site, Pol2-TAP was already enriched downstream of the break 15 minutes after HO induction, indicating the existence of RF stalling. Interestingly, 60 minutes after HO induction, there was still a clear Pol2 enrichment downstream but not upstream of the mini-*HOr* site. This was probably due to the presence of the centromere that delays the passage of the RF, as previously shown for chromosome III [29]. Indeed, the centromere slowed fork progression regardless of the *HO* site, as we confirmed by 2D-gel electrophoresis analysis in an α-factor synchronized culture of cells that were released in media with 20 mM HU (S4 Fig).

Similar to DNA polymerase 2, Rrm3 accumulation at specific DNA sites has also been used to identify RF pauses or stalls [9–11]. Consistently, Rrm3 enrichment resembled that of DNA Pol2 and was already observed downstream of the DNA break 15 minutes after HO endonuclease induction (Fig 6B). In contrast, Rad52 accumulated on both sides of the break after HO endonuclease induction (Fig 6B). These results support the conclusion that Rrm3 is recruited to the DNA break, together with the RF.

## Discussion

We studied the role of Rrm3 upon fork breakage in two situations that mimic the appearance of DSBs as a consequence of replication failures; this is, when an advancing replisome encounters a single stranded DNA break. We specifically induced such single stranded DNA breaks by the partial cleavage of the HO endonuclease taking advantage of an inefficient HO cleavage site that allows the cleavage of only one of the sister chromatids [6] or by the use of the *rad3-102* mutation that impairs NER after the step of endonuclease cleavage [17]. Using different genetic recombination assays we observed a defect in *rrm3*Δ cells only after HO-induced cleavage but not in spontaneous conditions (Fig 1). Noted was a clear decrease in the efficiency of SCE (Fig 4) but there was no role for Rrm3 in BIR (Fig 3), the latter in agreement with previous reports [25]). Both genetic and molecular tools have allowed us to uncover a role for Rrm3 in the repair of broken forks. Unlike most eukaryotes, *Saccharomyces cerevisiae* encodes two members of the conserved Pif1 family of DNA helicases: Rrm3 and Pif1 [30]. Therefore, it is likely that PIF1, the only member of the Pif1 helicase family in humans, works in the repair of broken forks to prevent genomic instability. Consistent with this view, mutations in human PIF1 are associated with increased cancer risk [31].

In agreement with yeast Pif1 and Rrm3 having many non-overlapping functions [32], the role we observe in SCE (Fig 4) is specific for Rrm3 and not observed in the other *Saccharomyces cerevisiae* member of the conserved Pif1 family of DNA helicases, Pif1, which is involved in BIR [25], or the Tof1 factor, involved in fork stabilization [33] (S3 Fig). Given that our SCE assay is based on a 10.5-Kb plasmid with a single replication origin (Fig 4), the replication-born one-ended DSBs would be converted into a two-ended DSB as the second and convergent fork reaches the site. In agreement, 15 minutes after the induction of HO, Rad52 enrichment was detected at both sides of the DSB (Fig 6). It is worth mentioning that the situation could be different at some chromosomal sites, where the adjacent fired origin is distant and the DSB remains one-ended for a longer time, although the requirement for BIR at the chromosome has been reported to be frequently suppressed by the convergent fork as well [34].

Importantly, the role of Rrm3 in DSB repair is specific for replication-born breaks, since we observed that the absence of Rrm3 does not lead to any defect in the repair of an enzymatically driven DSB at a full *HO* site or a double-stranded DNA gap generated by restriction endonuclease cleavage (Fig 2). Whether this is the main function of Rrm3 and whether the Rrm3 helicase activity is required in repair remains to be established.

In agreement with a positive role for Rrm3 in the repair of replication-dependent damage, Rrm3 accumulates at replication-born DSBs (Fig 6) and localizes at natural replication pausing sites together with the Smc5/6 complex [35], previously reported to promote sister-chromatid exchange [36]. Interestingly, *rrm3*Δ has a negative genetic interaction with *smc6* mutants that was first detected by high throughput screenings [37]. More recently, *rrm3*Δ has been shown to be lethal under low levels of Smc5 in G2 and this is likely dependent on Rad51 [35]. These genetic interactions might possibly be explained by the role of both Rrm3 (Fig 4) and the Smc5/6 complex [36] in the HR repair of replication-born DSBs with the sister chromatid.

The defective SCE observed in *rrm3*Δ cells could be due to a defective choice of the sister chromatid as a template for HR repair of DNA breaks arising during replication, as is thought to be the case for *smc5/6* mutants [36]. In agreement, *rrm3*Δ is known to accumulate gross chromosomal rearrangements [38] and a study that pinpointed *rrm3*Δ in a screen for mutants that accumulate Rad52 foci, detected a specific increase in the recombination with the homologous chromosome in *rrm3*Δ diploid cells [23].

However, non-sister templates cannot always compensate for the sister chromatid and the absence of Rrm3 seems to have an important impact on cell survival in the case of fork breakage (Fig 5). We have observed a genetic interaction of *rrm3*Δ with the *rad3-102* mutation (Fig 5, S2 Fig, [17]). Thus, indicating that, in the absence of Rrm3, the presence of replication-born DSBs in the chromosome leads to cell death, at least when RF progression is compromised. In agreement, the presence of an intact checkpoint is known to be essential for the survival of *rrm3*Δ cells [15]. Paradoxically, Rrm3 and Pif1 are detrimental to the integrity of replicating chromosomes under replication stress in the absence of the checkpoint protein Rad53 [39]. In checkpoint-deficient cells, RFs are known to arrest irreversibly upon replication stress leading to cell death [40–42]. Therefore, the action of Rrm3 for the repair of broken forks (Fig 4), which seems essential for survival in checkpoint-proficient cells (Fig 5), could be, however, detrimental in the case of an irreversible fork arrest. Consistent with this dual role, both Rrm3 deletion and overexpression lead to increased Rad52 foci and camptothecin hypersensitivity [23, 28, 43].

We propose an alternative functional explanation for the reported accumulation of Rrm3 at highly transcribed regions [13] and for the role for Rrm3 in RF progression through described transcription-dependent pausing sites [14]. Our demonstration that Rrm3 is required for the repair of replication-born DSBs would support that the accumulation of Rrm3 at sites of active transcription [13] is not just a consequence of Rrm3 being a replisome component but might also reflect an active role of Rrm3 in the repair of broken forks.

## Materials and methods

### Strains and plasmids

Yeast strains used in this work are listed in S1 Table. *leu2-k* strains were obtained from the pop up of the *URA3-ADE2* cassette of *leu2-k*::*URA3-ADE2*::*leu2-k* strains in FOA. Plasmids *pRS316-TINV*, *pCM189-L2HOr*, *pCM189-LEU2*, *pRS313-GALHO* and *pRS315-GALHO* were described previously [5, 7, 21].

### Physical analysis of SCE

SCE assays were carried out essentially as described [5, 21]. Briefly, cells carrying *pRS316-TINV* plasmid were grown to mid-log phase in synthetic complete (SC) medium -Ura 3% glycerol 2% lactate; then, galactose (2%) was added to induce HO expression. Samples were collected at different time points and DNA was purified, digested with *Spe*I-*Xho*I, and analysed by Southern blot using Hybond N+ (GE Healthcare) membranes. A ^32^P-labeled 0.22-kb *LEU2* probe was obtained by PCR using the primers 5´-GTTCCACTTCCAGATGAGGC-3´ and 5´-TTAGCAAATTGTGGCTTGA-3´. Each experiment was done in triplicate and quantified using a PhosphorImager Fujifilm FLA-5100 and the ImageGauge program. Only a representative experiment is shown.

### Genetic analysis of HR induced by HO-mediated ssDNA breaks

The analysis of HR induced by HO-mediated ssDNA breaks was performed as described [20]. Briefly, mid-log phase yeast cells carrying the HO gene under the control of *GAL1* were obtained from SC-3% glycerol-2% lactate liquid cultures and split into two halves. One-half was maintained in liquid SC-3% glycerol/2% lactate (no HO expression) and the other was cultured in SC-2% galactose (HO expression) for the indicated time. Doxycycline was also added at 5 μg/ml to the media to repress transcription from the *TET* promoter. Recombinants were selected on SC-Leu-Ura containing 2% glucose. The plasmid-chromosome system *leu2-HOr/leu2-k* [22] was used to analyse HR (gene conversion). In all cases, HR frequencies are the mean values of three transformants. For each transformant, HR is the median of six independent yeast colonies as previously described [21].

### Genetic analysis of repair of plasmid double-stranded DNA gaps

Plasmid *pCM189-L2HOr* was digested with the *Mfe*I enzyme and the linear DNA was gel purified. Transformation into *leu2-k* host strains was performed by the lithium acetate transformation method with 200 ng of gapped plasmid (gap repair substrate) or 200 ng of uncut plasmid (transformation efficiency control) in the presence of 50 μg of denatured salmon sperm DNA as carrier DNA. Transformed cells were diluted and plated onto SC−Leu−Ura media. The HR frequency was calculated as the number of Leu+ Ura+ recombinants per microgram of transformed gapped plasmid divided by the total number of Ura+ transformants per microgram of transformed uncut plasmid as previously described for analogous systems [44].

### Molecular analysis of DSB repair

The efficiency of DSB repair was analysed as the capacity of re-joining of an HO-induced DSB at the *MAT* locus as previously described [45]. For this, cells carrying the *pRS313-GALHO* plasmid were grown at 30°C to mid-log phase in SC-Ura 3% glycerol 2% lactate. HO expression was induced for 2 hours in galactose and then repressed by glucose addition before colleting the samples at the indicated time points. DNA was extracted from different samples purified, digested with *Hind*III, and analysed by Southern-blot using a specific *MAT* probe obtained by PCR amplification with primers 5’-ACAAGGAAGCTGACTGTGGA-3´ and 5´-CGCACACCATTTCCTACTGG-3´. DSB signals were calculated for each time-point as the intensity of the 3.2-Kb cleaved band with respect to the signal of the 3.2- and 4.1-Kb bands. The percentage of DSB was similar in both strains and thus normalized in each experiment to the time-point 0 of the corresponding strain. Signals were quantified using a PhosphorImager Fujifilm FLA-5100 and the ImageGauge program.

### Genetic determination of triparental BIR-mediated translocation frequencies

Triparental-BIR frequencies were determined as described [26]. Briefly, the indicated strains were grown at 30°C to mid-log phase in SC- 3% glycerol 2% lactate. HO endonuclease expression was induced by the addition of 2% galactose. After 24 hours of incubation in the presence of galactose, appropriate dilutions were plated on galactose media to determine the total cell number and on galactose media without leucine to determine the number of recombinants.

### Molecular detection of single BIR intermediates

The single BIR reaction was detected as described [26]. The indicated strains were grown at 30°C to mid-log phase in SC- 3% glycerol 2% lactate. HO endonuclease expression was induced by the addition of 2% galactose. Genomic DNA samples were extracted at the indicated time points to perform PCR with the indicated primers (S2 Table) with MyTAQ DNA polymerase standard conditions. The PCR products amplified with primers p1 and p2 were quantified with ImageJ and normalised to those of the control PCR with primers p1 and p4.

### Chromatin immunoprecipitation (ChIP) analysis

For ChIP experiments, exponentially growing cells were cultured in SC medium containing 3% glycerol and 2% lactate. The culture was then split in two; one half was supplemented with 2% glucose (-HO) and the other half with 2% galactose (+HO). Samples were collected at the indicated time points, and ChIP assays were performed essentially as described [46] with monoclonal Anti-FLAG M2 antibody (Sigma F1804), Dynabeads Protein A (Invitrogen) for Rrm3-FLAG and Rad52-FLAG immunoprecipitation and Ig-Sepharose for Pol2-TAP. The GFX purification system (Amersham) was used for the last DNA purification step. For each experiment, the DNA ratios in the different *leu2-HOr* regions were calculated from the amount of DNA in these regions relative to that in the Ampicillin resistance region. The relative abundance of each DNA fragment was calculated normalizing IP/input ratios as previously described [46]. In all cases, ChIPs were performed from three independent cultures, and quantitative PCRs were repeated three times for each culture. The primers used are shown in S2 Table. Medians and SD of three independent experiments are shown.

### Cell cycle synchronization and flow cytometry

*bar1*Δ strains were used for cell cycle synchronization to prevent adaptation to α-factor. Cells were arrested in the G1 stage with 5 μg/ml α-factor mating pheromone and were released into SC medium to allow synchronous progression into the S phase. Approximately 10^7^ cells were collected at each of the indicated time points postrelease from α-factor arrest and processed for flow-cytometry analysis. Samples were processed as described previously [17] and cell cycle distribution was determined using a FACSCalibur system (Becton-Dickinson).

### Analysis of RF progression by 2-D gel electrophoresis

The indicated *bar1*Δ strains were arrested with α-factor and released into minimal medium containing 20 mM hydroxyurea for 30 min prior to DNA extraction. DNA extraction was performed with the cetyltrimethylammonium bromide method, and neutral-neutral 2-D gel electrophoresis was performed as previously described [17]. Probes for 2D gel analyses were obtained by PCR amplification with primers 5’ CAAGAAGGAGAAAAAGGAGG-3’ and 5´-CGCCTTTGAGTGAGCTGATA-3’ for L fragment and primers 5’-AATGTCAACATGGCGGTAAT-3’ and 5’- CGAGCTCGACTTTCACTTTT-3’ for R fragment. Signals were quantified using a PhosphorImager Fujifilm FLA-5100 and the ImageGauge program.

## Supporting information

S1 Fig Growth curves. (A) Growth curve of wild-type and *rrm3Δ* cells cultures in the course of the experiment depicted in Fig 2. The numbers plotted on the graph correspond to the number of cells (x10^3^) in each time point for each strain. (B) Growth curve of wild-type and *rrm3Δ* cells cultures in the course of the experiment depicted in in Fig 4. The numbers plotted on the graph correspond to the number of cells (x10^3^) in each time point and for each strain.
(TIF)

S2 Fig Genetic interaction of *rrm3Δ* with *rad3-102* in the presence of different DNA damaging agents. Sensitivity to 4-NQO, HU, MMS and UV of the indicated strains was tested by 10-fold serial dilutions of exponentially growing cultures.
(TIF)

S3 Fig Physical analysis of SCE in *pif1Δ*, *tof1Δ* and *rrm3Δ*. (A) HO-induced formation of DSB and SCE intermediates in isogenic BY wild-type and indicated mutant cells transformed with the *pRS315-GALHO* plasmid incubated in galactose for the indicated time points. Other details as in Fig 4. (B) Quantification of DSBs (1.4 Kb plus 2.4 Kb bands) and SCE (4.7 Kb band) relative to the total DNA. The average and SEM of two independent experiments is shown.
(TIF)

S4 Fig RF progression analysis by 2D-gel electrophoresis. (A) Top, schematic representation of the *pCM189-LEU2* analysed plasmid showing the position of centromere, ARS and the relevant probes (R and L). Bottom, restriction fragments analysed by two-dimensional-gel electrophoresis and schematic representation of the migration pattern of single Y molecules by two-dimensional-gel electrophoresis. (B) Analysis of RF progression through R and L fragments at LEU2 gene from DNA samples digested with BglII and BamHI (R Fragment) or BglII and PstI (L Fragment) in wild-type cells. Cells were synchronized in G1 with α factor and monitored at different time points after release in 20 mM HU. (C) Quantification of the replicative intermediates. The ratio of the signal in the descending Y arc versus the total replicating molecules is plotted. (D) FACS profiles from a representative experiment.
(TIF)

S1 Table Strains used for this study. (XLSX)

S2 Table Primers used for the chromatin immunoprecipitation analysis. (XLSX)
