# Supplementary figures and images for: A new role for Rrm3 in repair of replication-born DNA breakage by sister chromatid recombination
Source: PLoS Genet. 2017 May 5;13(5):e1006781. doi: 10.1371/journal.pgen.1006781 (PMC5438189; doi:10.1371/journal.pgen.1006781)

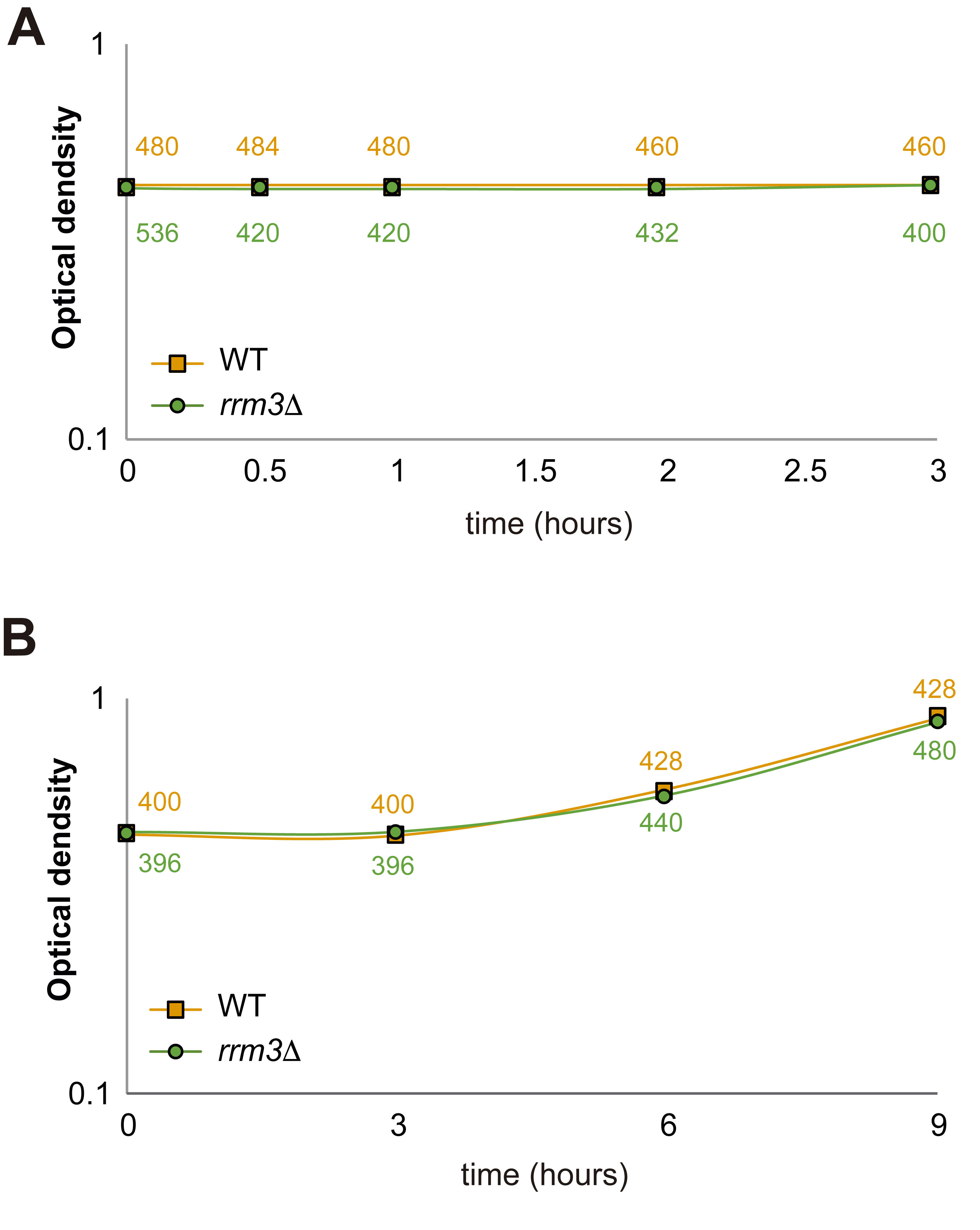

Supplement: S1 Fig — (A) Growth curve of wild-type and rrm3Δ cells cultures in the course of the experiment depicted in Fig 2. The numbers plotted on the graph correspond to the number of cells (x103) in each time point for each strain. (B) Growth curve of wild-type and rrm3Δ cells cultures in the course of the experiment depicted in in Fig 4. The numbers plotted on the graph correspond to the number of cells (x103) in each time point and for each strain. (TIF) [file pgen.1006781.s001.tif]

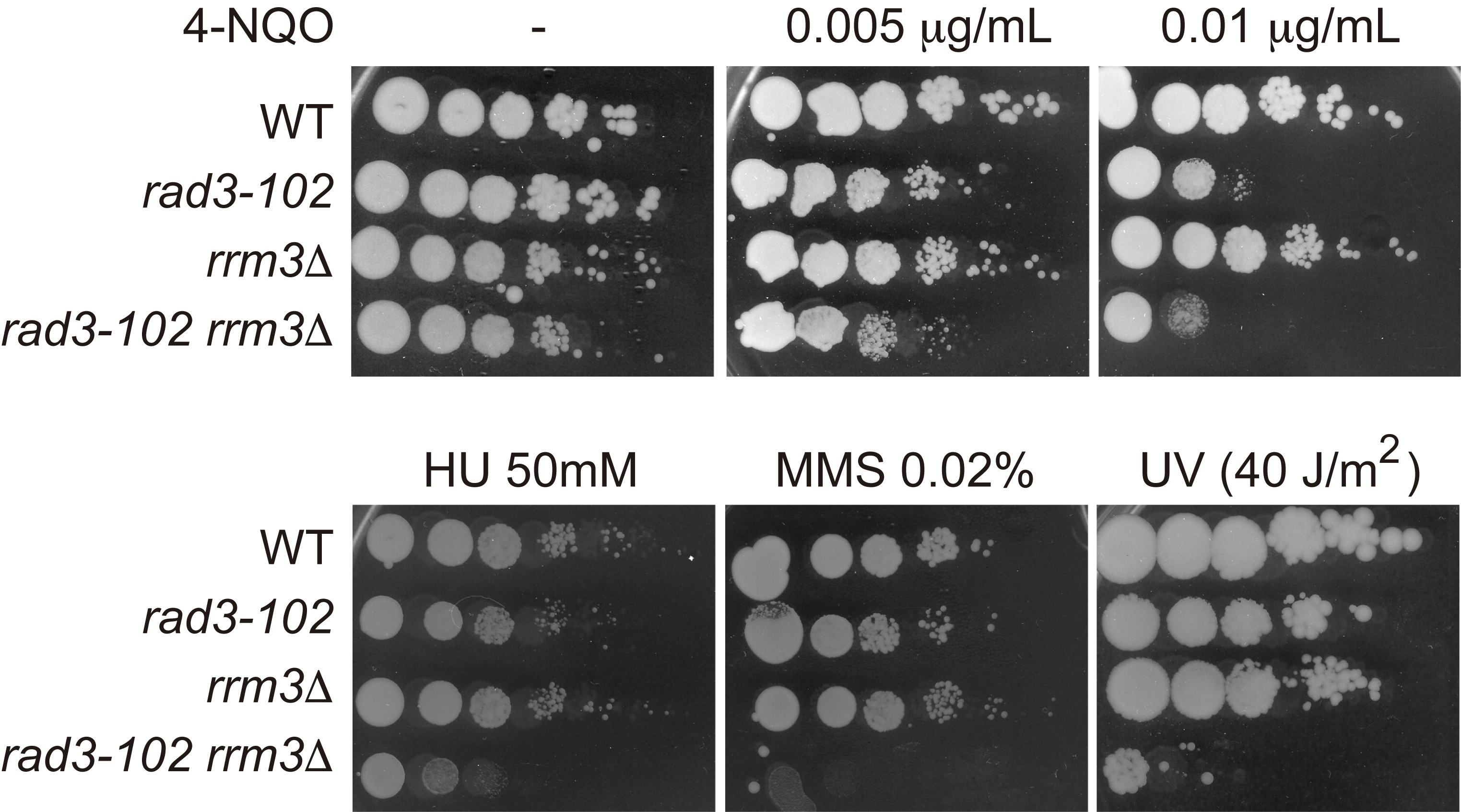

Supplement: S2 Fig — Sensitivity to 4-NQO, HU, MMS and UV of the indicated strains was tested by 10-fold serial dilutions of exponentially growing cultures. (TIF) [file pgen.1006781.s002.tif]

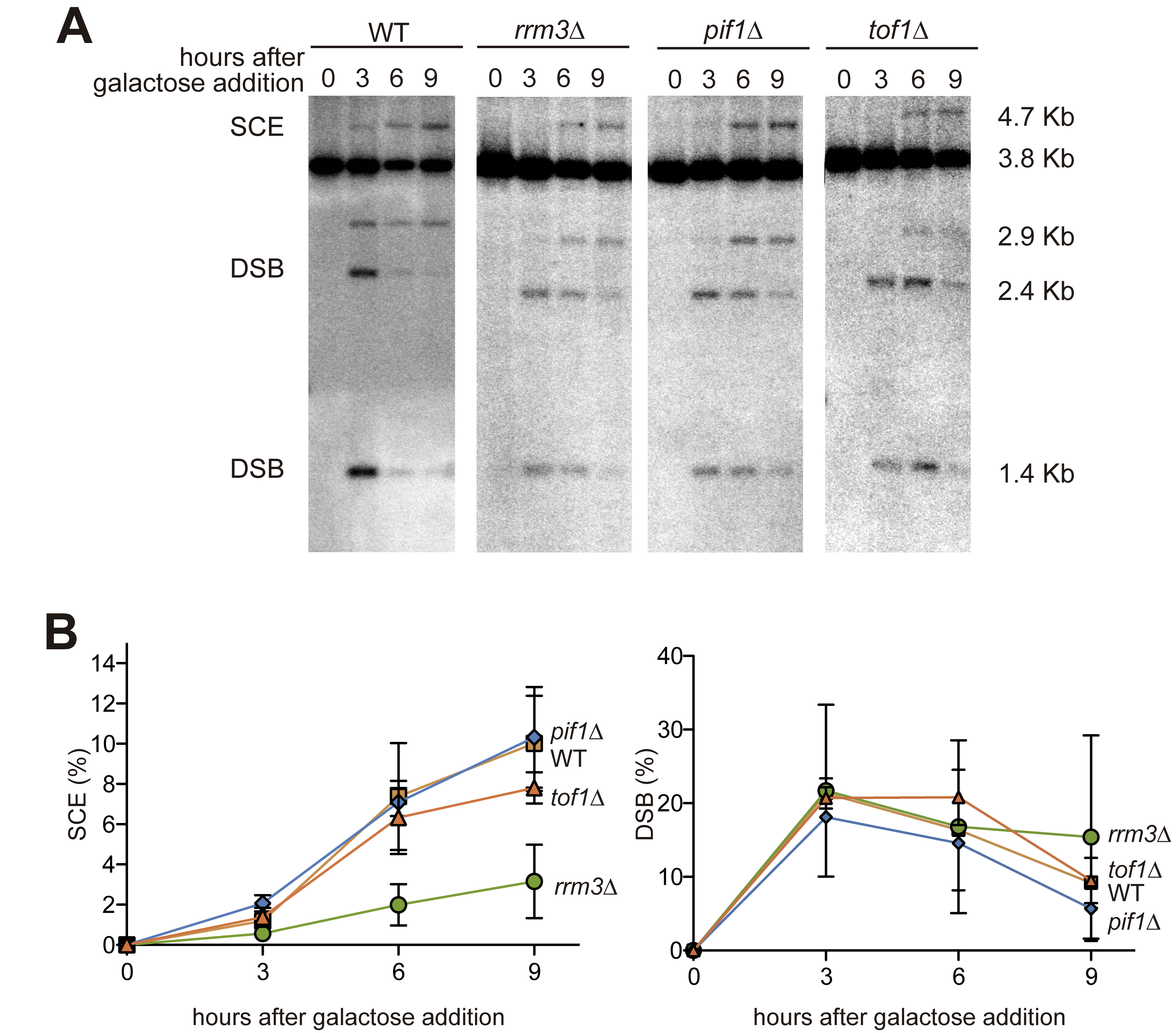

Supplement: S3 Fig — (A) HO-induced formation of DSB and SCE intermediates in isogenic BY wild-type and indicated mutant cells transformed with the pRS315-GALHO plasmid incubated in galactose for the indicated time points. Other details as in Fig 4. (B) Quantification of DSBs (1.4 Kb plus 2.4 Kb bands) and SCE (4.7 Kb band) relative to the total DNA. The average and SEM of two independent experiments is shown. (TIF) [file pgen.1006781.s003.tif]

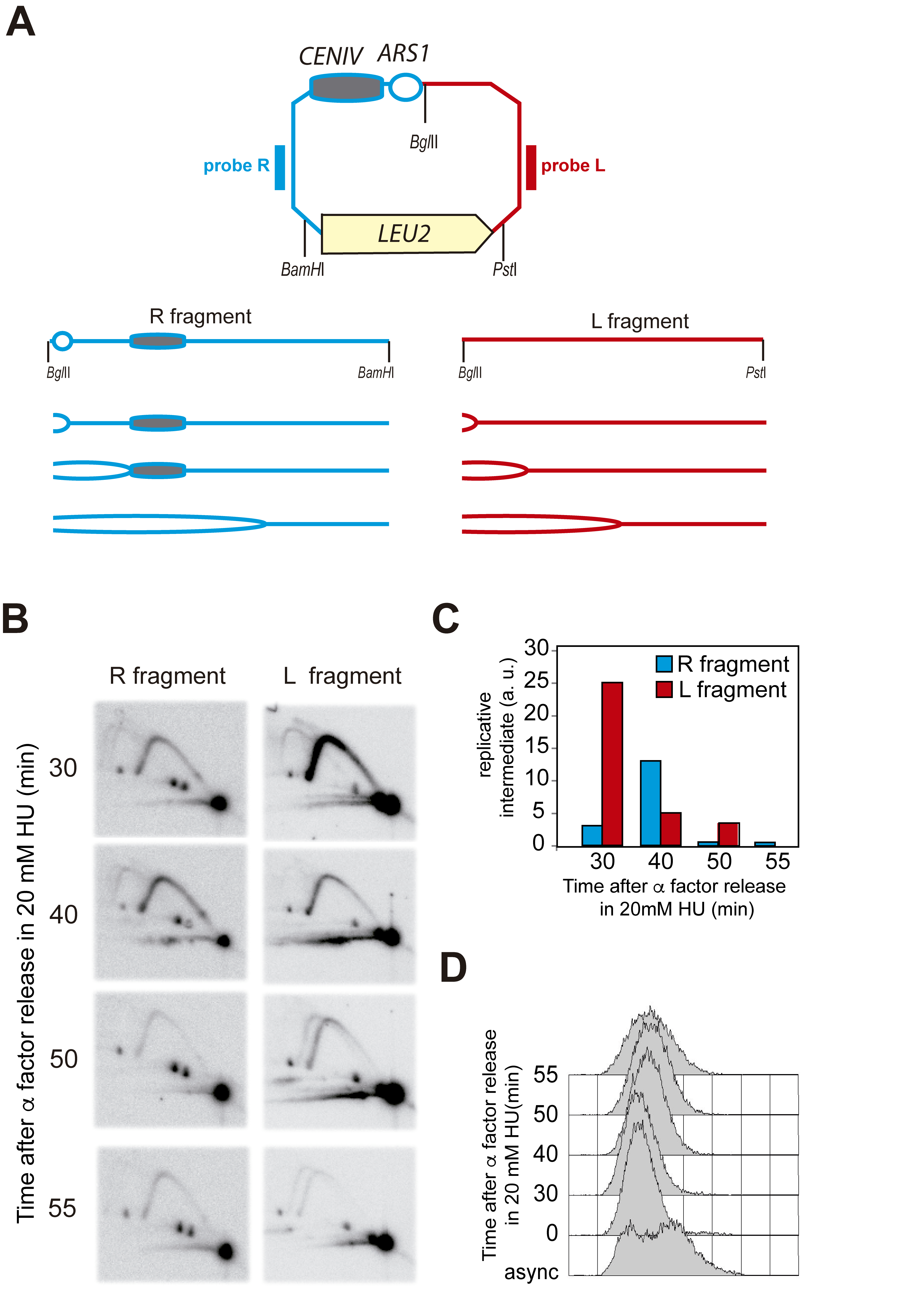

Supplement: S4 Fig — (A) Top, schematic representation of the pCM189-LEU2 analysed plasmid showing the position of centromere, ARS and the relevant probes (R and L). Bottom, restriction fragments analysed by two-dimensional-gel electrophoresis and schematic representation of the migration pattern of single Y molecules by two-dimensional-gel electrophoresis. (B) Analysis of RF progression through R and L fragments at LEU2 gene from DNA samples digested with BglII and BamHI (R Fragment) or BglII and PstI (L Fragment) in wild-type cells. Cells were synchronized in G1 with α factor and monitored at different time points after release in 20 mM HU. (C) Quantification of the replicative intermediates. The ratio of the signal in the descending Y arc versus the total replicating molecules is plotted. (D) FACS profiles from a representative experiment. (TIF) [file pgen.1006781.s004.tif]
